# Disease-Specific Survival of Type I and Type II Epithelial Ovarian Cancers—Stage Challenges Categorical Assignments of Indolence & Aggressiveness

**DOI:** 10.3390/diagnostics10020056

**Published:** 2020-01-21

**Authors:** Edward J. Pavlik, Christopher Smith, Taylor S. Dennis, Elizabeth Harvey, Bin Huang, Quan Chen, Dava West Piecoro, Brian T. Burgess, Anthony McDowell, Justin Gorski, Lauren A. Baldwin, Rachel W. Miller, Christopher P. DeSimone, Charles Dietrich, Holly H. Gallion, Frederick R. Ueland, John R. van Nagell

**Affiliations:** 1 Department of Obstetrics & Gynecology, University of Kentucky, Lexington, KY 40536, USA; christophergsmith@uky.edu (C.S.); brian.burgess@uky.edu (B.T.B.); anmcdowe@gmail.com (A.M.); justin.gorski@uky.edu (J.G.); labald1@email.uky.edu (L.A.B.); raware00@email.uky.edu (R.W.M.); cpdesi00@uky.edu (C.P.D.); charles.dietrich@uky.edu (C.D.III); Holly.Gallion1@uky.edu (H.H.G.); fuela0@uky.edu (F.R.U.); jrvann2@email.uky.edu (J.R.v.N.J.); 2 Division of Gynecologic Oncology, Markey Cancer Center, Lexington, KY 40536, USA; 3 Denison University, Granville, OH 43023, USA; taylor.dennis6@gmail.com; 4 Department of Obstetrics & Gynecology, Dartmouth-Hitchcock Medical Center, Lebanon, NH 43023, USA; Elizabeth.A.Harvey@hitchcock.org; 5 Division of Cancer Biostatistics, College of Public Health & Biostatistics Shared Resource Facility, Markey Cancer Center, University of Kentucky, Lexington, KY 40536, USA; bhuang@kcr.uky.edu (B.H.); quan@kcr.uky.edu (Q.C.); 6 Department of Pathology and the Markey Cancer Center, Lexington, KY 40536, USA; Dava.west@uky.edu

**Keywords:** epithelial ovarian cancer, survival, Type I & Type II, histo-types, grade, stage

## Abstract

Epithelial ovarian cancers (EOC) consist of several sub-types based on histology, clinical, molecular and epidemiological features that are termed “histo-types”, which can be categorized into less aggressive Type I and more aggressive Type II malignancies. This investigation evaluated the disease-specific survival (DSS) of women with Type I and II EOC using histo-type, grade, and stage. A total of 47,789 EOC cases were identified in the National Cancer Institute’s Surveillance, Epidemiology, and End Results (SEER) data. Survival analysis and log rank test were performed to identify a 2-tiered classification (grade 1 vs. grade 2 & 3) for serous EOC. DSS of early stage serous EOC for grade 2 was significantly different from grade 3 indicating that a 2-tier classification for serous EOC applied only to late stage. DSS of Type I EOC was much better than Type II. However, DSS was 33–52% lower with late stage Type I than with early stage Type I indicating that Type I ovarian cancers should not be considered indolent. Early stage Type II EOC had much better DSS than late stage Type II stressing that stage has a large role in survival of both Type I and II EOC.

## 1. Introduction

In 2020 21,750 new cases of epithelial ovarian cancer (EOC) will be diagnosed in the United States, accounting for 13,940 deaths [1]. EOC kills more women than all other gynecologic malignancies combined, representing the fifth most frequent cause of death from cancer for women and accounts for 7.7 deaths per 100,000 women or 5.1% of all cancer deaths [2,3]. While the average lifetime risk of developing EOC is 1.3% or roughly 1 in 78 women [4] which is much lower than breast cancer, EOC has a death-to-incidence ratio that is 3–4 times greater than breast cancer [2,5,6].

EOCs are a heterogeneous group of malignancies consisting of dissimilar cell-types and different biological behaviors. Some EOC classified as ovarian in origin are now thought to arise from the fallopian tubal epithelium [7,8,9,10]. EOC remains the most common cell type accounting for >85 percent of cases [3,11]. Traditionally, EOC is classified into histo-types as serous, endometrioid, mucinous, clear cell, carcinosarcoma, or undifferentiated carcinoma [12,13,14].

EOC has been classified by histological subtype since the 1930′s and 1940′s, in a system that has been adopted and updated by the World Health Organization [15]. The classification paradigm has been modernized to include not only tumor morphology, but immunohistochemical and molecular characterizations to differentiate Type I or Type II EOC [16,17,18]. Clinically, Type I tumors are thought to occur as large, unilateral, cystic neoplasms confined to the ovary. They are perceived as typically low-grade, with indolent or less aggressive clinical behavior and a more favorable prognosis. It has been hypothesized that Type I neoplasms develop from benign extra-ovarian lesions that embed in the ovary, and ultimately undergo malignant transformation. In contrast, Type II ovarian tumors are high-grade, can involve both ovaries, exhibit aggressive biological behavior, tend to present in advanced stage, and have poorer outcomes. A popular speculation is that some Type II EOC develop from abnormal fallopian tube epithelium called serous tubal intra-epithelial lesions (STIL) that progress to serous tubal intra-epithelial carcinomas (STIC). Type II EOC includes high-grade serous carcinoma, high-grade endometrioid carcinoma, carcinosarcoma, and undifferentiated carcinoma [9,10,11,19,20,21].

In the present study we have examined the disease-specific survival (DSS) characteristics of Type I and Type II EOCs through their underlying histologic subtypes and relationships to grade and stage.

## 2. Materials and Methods

EOC cases were identified through the National Cancer Institute’s Surveillance, Epidemiology, and End Results (SEER) program, which collects data on every cancer case reported from 20 geographic areas of the United States (approximately 30% of the population) that represent the underlying demographics for the entire US population [22]. Data from reports meeting the following criteria were extracted from the SEER 18 registries in the November 2016 data submission covering the period 1995–2015 released in April 2018: EOC histo-types (mucinous, clear cell, endometrioid, serous carcinomas, carcinosarcomas, and undifferentiated carcinomas) with cause of death, grade and stage in order to evaluate DSS for Type I and Type II EOCs. The 1995–2015 time-period was chosen for examination because after 1995, most patients were expected to be surgically staged and receive platinum/taxane chemotherapy [23]. Exclusions were based on the insufficient histo-type designation, absence of cause of death or absence of pathology evaluation (grade and stage) or due to insufficient numbers of subjects for Kaplan–Meier analysis of DSS (Brenner tumors). Designation of only “carcinoma”, NOS or mixed histo-type were excluded. Based on the 10-year DSS survival rates, breakpoints were identified to categorize early and late stage EOC subtypes into similar risk groups then the groupings were validated using pair-wised and multiple log-rank tests. SEER codes for primary EOC histo-types as identified below were selected as the most unambiguous representation of each histo-type. SEER grade 4 cases were re-classified and combined with grade 3 cases to agree with International Federation of Gynecology and Obstetrics (FIGO) classification. Type I EOC consisted of: all grades of mucinous carcinomas (International Classification of Diseases, 10th Revision (ICD 10) SEER code 8470/3, 8471/3, 8480/3, 8481/3, 8482/3), all grades of clear cell carcinomas (ICD 10 SEER code 8310/3), grades 1 & 2 of endometrioid carcinomas (ICD 10 SEER code 8380/3,), and grade 1 serous carcinomas (ICD 10 SEER code 8441/3, 8460/3, 8461/3). Type II ovarian carcinomas consisted of: grade 3 endometrioid carcinomas (ICD 10 SEER code 8380/3), grades 2–3 of serous carcinomas (ICD 10 SEER code 8441/3, 8460/3, 8461/3), carcinosarcomas (ICD 10 SEER codes 8950/3, 8951/3 & 8980/3, high grade by definition), and undifferentiated carcinoma (ICD 10 SEER code 8020/3, high grade by definition). A total of 47,789 records met the EOC histo-type criteria out of an inclusion set of 105,128 EOC SEER records. 40,587 cases were first primary or only primary cases, and of these 35,901 were accompanied by grade and stage information.

## 3. Results

### 3.1. Survival Analyses of Individual Ovarian Cancer Histo-Types Related to Intrinsic Differentiation

**Figure 1:**
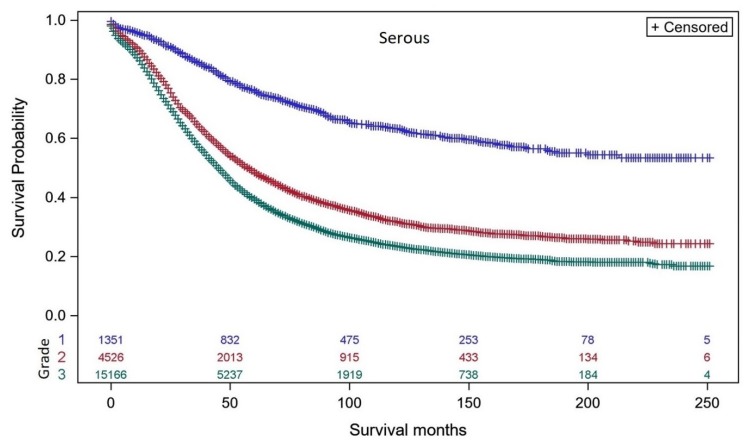
Kaplan–Meier ovarian cancer-specific survival showing number of women at risk according to grade of serous EOC (8441/3, 8460/3, 8461/3). Women with grade 1 well differentiated serous EOC (blue curve, *n* = 1,351; 50, 100 & 200-month survival is 80.0%, 66.2%, 55.0%), grade 2 moderately differentiated (red curve, *n* = 4,526; 50, 100 & 200-month survival is 55.8%, 36.5%, 25.7%), and grade 3 undifferentiated (green curve, *n* = 15,166; 50, 100 & 200-month survival is 48.3%, 27.7%, 19.0%). The number of women remaining at different survival points is shown for well differentiated screened (blue), moderately differentiated (red), undifferentiated (green) cases. Log-rank test *p* < 0.001.

**Figure 2:**
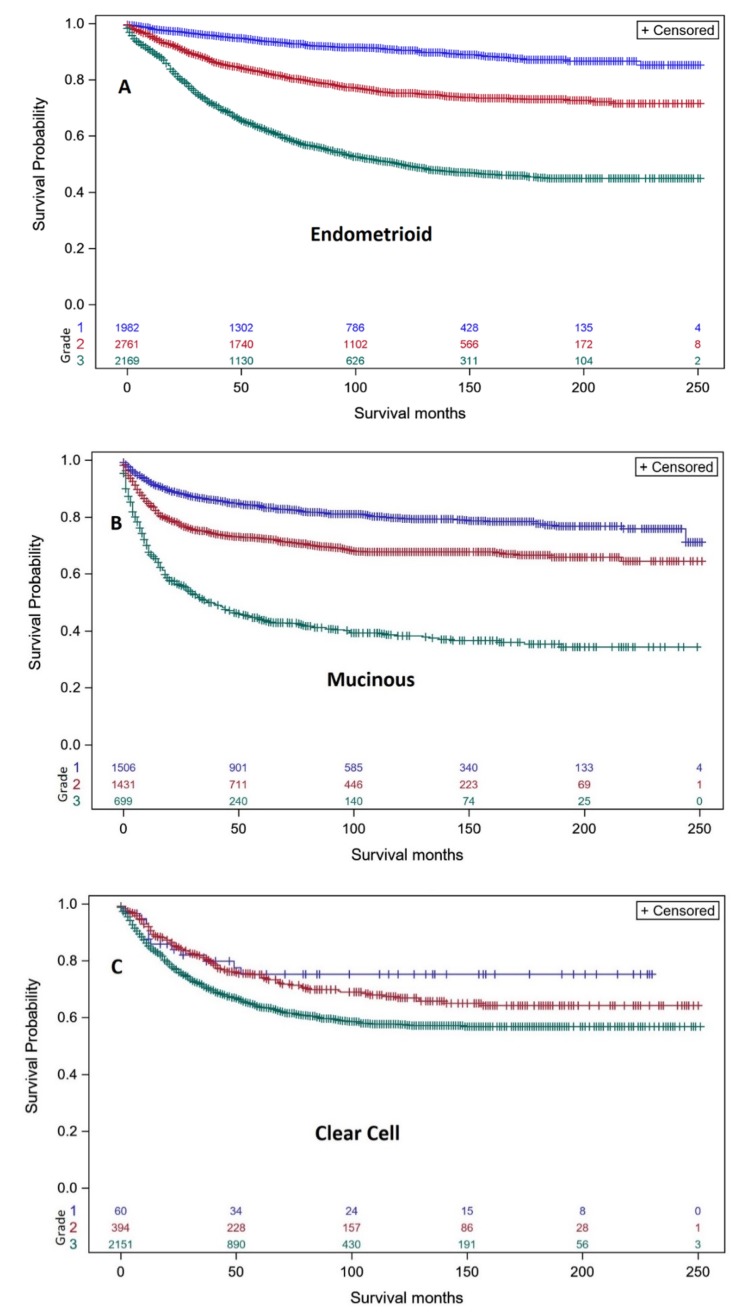
Kaplan–Meier ovarian cancer-specific survival showing number of women at risk according to grade of endometrioid EOC, mucinous carcinoma and clear cell EOC. (**A**). Women with endometrioid EOC (8380/3) that was grade 1 well differentiated (blue curve, *n* = 1982; 50, 100 & 200-month survival is 94.8%, 91.4%, 86.5%), grade 2 moderately differentiated (red curve, *n* = 2761; 50, 100 & 200-month survival is 84.3%, 77.1%, 71.7%), and grade 3 undifferentiated (green curve, *n* = 2169; 50, 100 & 200-month survival is 66.4%, 52.9%, 44.4%). Log-rank test *p* < 0.001. (**B**). Women with mucinous EOC (8470/3, 8471/3, 8480/3, 8481/3, 8482/3) that was grade 1 well differentiated (blue curve, *n* = 1506; 50, 100 & 200-month survival is 84.5%, 80.7%, 76.4%, grade 2 moderately differentiated (red curve, *n* = 1431; 50, 100 & 200-month survival is 72.1%, 67.7%, 65.8%), and grade 3 undifferentiated (green curve, *n* = 699; 50, 100 & 200-month survival is 46.5%, 39.7%, 35.2%). Log-rank test *p* < 0.001. (**C**). Women with clear cell EOC (8310/3) that was grade 1 well differentiated (blue curve, *n* = 60; 50, 100 & 200-month survival is 77.4%, 74.7%, 74.7%), grade 2 moderately differentiated (red curve, *n* = 394; 50, 100 & 200-month survival is 76.2%, 69.0%, 63.3%), and grade 3 undifferentiated (green curve, *n* = 2151; 50, 100 & 200-month survival is 67.1%, 58.6%, 56.3%). The number of women remaining at different survival points is shown for well differentiated screened (blue), moderately differentiated (red), undifferentiated (green). Log-rank test *p* < 0.001.

**Figure 3:**
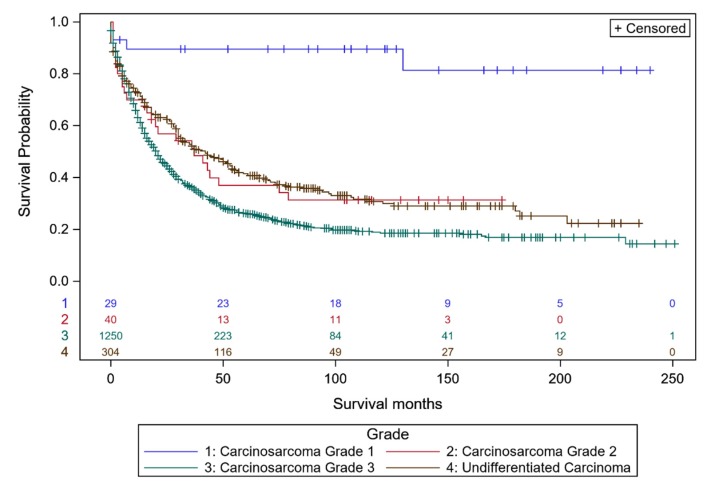
Kaplan–Meier ovarian cancer-specific survival showing number of women at risk according to grade of ovarian carcinosarcoma and undifferentiated carcinoma. Women with ovarian carcinosarcoma (8950/3, 8951/3 & 8980/3) that was grade 1 well differentiated (blue curve, *n* = 29; 50, 100 & 200-month survival is 89.1.0%, 89.1%, 81.7%), grade 2 moderately differentiated (red curve, *n* = 40; 50, 100 & 200-month survival is 37.7%, 31.9%, 31.9%), and grade 3 undifferentiated (green curve, *n* = 1250; 50, 100 & 200-month survival is 31.8%, 21.8%, 18.9%). Log-rank test *p* < 0.001. Women with undifferentiated EOC (8020/3) that was grade 3 by definition (black curve, *n* = 304; 50, 100 & 200-month survival is 46.9%, 32.9%, 25.8%).

Well differentiated (grade 1: blue curve) serous EOC had much better DSS than moderately differentiated (grade 2: red curve) or poorly differentiated (grade 3: green curve) serous EOC (Figure 1). The separation in DSS between grade 1 and grade 2–3 serous EOC supports a two tier classification [24,25,26] of the serous histo-types (low grade = grade 1 vs. high grade = grade 2–3). In contrast, the DSS of women with moderately differentiated grade 2 endometrioid carcinomas (Figure 2A: red curve) was closer to DSS of women with well differentiated grade 1 endometrioid carcinomas (Figure 2A: blue curve). Importantly, DSS of poorly differentiated (grade 3) endometrioid carcinoma was superior to DSS for poorly differentiated (grade 3) serous EOC (44% vs. 19% at 200–250 months). DSS of moderately differentiated (grade 2) mucinous ovarian carcinoma (Figure 2B: red curve) was intermediate between well-differentiated (grade 1, blue curve) and poorly differentiated (grade 3) mucinous carcinoma (green curve) with DSS of poorly differentiated (grade 3) mucinous carcinoma better than poorly differentiated (grade 3) serous EOC (35% vs. 19% at 200–250 months), and less than DSS for poorly differentiated (grade 3) endometrioid carcinoma (35% vs. 44% at 200–250 months). DSS of women with moderately differentiated (grade 2) clear cell ovarian carcinomas (Figure 2C: red curve) was very similar to DSS of well-differentiated (grade 1) clear cell carcinomas (blue curve). Poorly differentiated (grade 3) clear cell carcinomas (green curve) demonstrated DSS better than poorly differentiated (grade 3) endometrioid carcinomas (56% vs. 44%) and better DSS than poorly differentiated (grade 3) serous EOC (55% vs. 19% at 200–250 months) or poorly differentiated mucinous ovarian carcinoma (55% vs. 35% at 200–250 months). DSS of women with moderately differentiated (grade 2) carcinosarcoma (Figure 3: red curve) was intermediate between well-differentiated (grade 1, blue curve) and poorly differentiated (grade 3) carcinosarcoma (green curve) with DSS of poorly differentiated grade 3 carcinosarcoma similar to poorly differentiated (grade 3) serous EOS (18.9% vs. 19% at 200–250 months), but less than for poorly differentiated (grade 3) endometrioid carcinoma (18.9% vs. 44% at 200–250 months), poorly differentiated (grade 3) mucinous carcinoma (18.9% vs. 35% at 200–250 months) or poorly differentiated (grade 3) clear cell carcinoma (18.9% vs. 56% at 200–250 months). DSS of women with undifferentiated carcinoma (Figure 3 brown curve) was similar to undifferentiated (grade 3) carcinosarcoma (26% vs. 18.9% at 200–250 months) and slightly greater than undifferentiated (grade 3) serous EOC (26% vs. 19% at 200–250 months).

### 3.2. Survival Analyses of Individual Ovarian Cancer Histo-Types Related to Differentiation and Extent of Disease

Ten year DSS was used to examine early (Stage I-II) and late stage disease (Stage III-V), as well as histologic grade (Table 1). For early stage serous EOC, grade 1 and grade 2 were statistically different as were grade 2 and grade 3 (*p* < 0.001). For late stage serous EOC, grade 1 and grade 2 were statistically different but grade 2 and grade 3 were not (*p* < 0.001). Thus, the 2-tier construct for serous EOC is most applicable to late stage disease where DSS for grades 2 & 3 are similar and statistically distinct from grade 1. Ten year DSS for late stage carcinosarcomas was significantly lower grade-by-grade than early stage (*p* < 0.001, Table 1). For between grade comparisons of late stage carcinosarcoma, DSS was worse for grade 2 in contrast to grade 1, while grade 3 DSS was significantly poorer than grade 2 (*p* < 0.001, Table 1) supporting the notion that DSS of all grades of late stage carcinosarcoma are similarly poor, and much poorer than DSS for all grades of early stage disease. For early stage disease of all histo-types, statistically significant decreases in DSS occurred as grade increased (Table 1). The findings in Table 1 demonstrate that DSS of early stage disease decreases with increasing grade, and is better than late stage DSS for all histo-types in grade-by-grade comparisons. Lastly, undifferentiated carcinomas are all grade 3 with better DSS observed for early stage disease. Undifferentiated carcinomas had a 10 year DSS that was not statistically different from the 10-year DSS for high grade serous carcinomas and carcinosarcomas. Sequential analysis of 10-year DSS for early stage disease reveals statistically significant groupings that are greater than 93%, 90%, 80–87%, 68–79%, 54–65% and 44% (carcinosarcoma grade 3), Table 2. Analysis of late stage disease identified 10-year DSS that ranged from 64% (Endometrioid grade 1) to 15% (Mucinous grade 3), Table 3. Taken together early stage disease is characterized by higher 10-year DSS in all histo-types with increasing grade accounting for poorer 10-year DSS in both early and late stage disease. The average decrease in 10-year DSS due to progression from early to late stage EOC was 46.2% for grade 1, 53.5% for grade 2 and 43.9% for grade 3 malignancies, and 46.9% overall grades and histo-types.

### 3.3. Analyses of Type I & II Ovarian Cancers

The differences that define Type I (green font) and Type 2 (red font) EOCs are highlighted in Table S1 of the Supplementary Material section. Type I EOCs have been regarded as indolent and Type II as aggressive with grade providing discrimination between the two types in ways that are specific to each EOC subtype. Low grades (grade 1 & 2) generally define Type I EOC (except for serous grade 2 carcinomas, undifferentiated carcinomas and carcinosarcomas) with high grade (grade 3) defining Type II ovarian carcinomas (except for clear cell, mucinous, transitional cell and malignant Brenner’s tumors). Each subtype is proposed to originate from unique precursors. Finally, different molecular markers have low or high expression in Type I vs. Type II ovarian carcinomas, but are rarely totally expressed (PTEN mutation, PIK3CA, KRAS mutation, BRAF mutation) or totally absent in either Type I or Type II EOCs.

**Figure 4:**
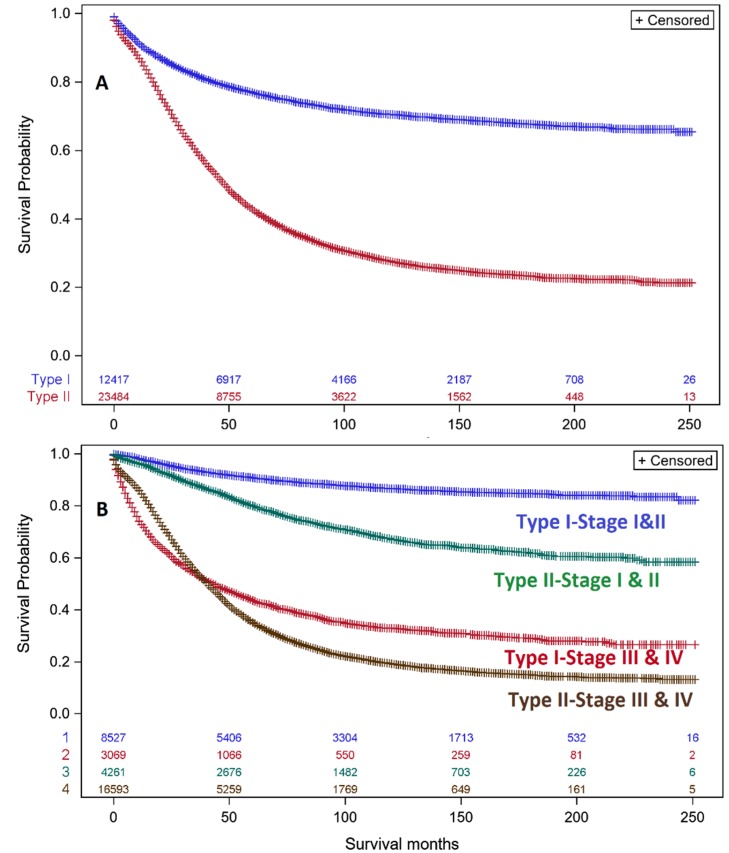
Kaplan–Meier survival of women with Type I & II EOC. (**A**). DSS of women with Type I EOC (blue curve, *n*= 12,417; 50, 100 & 200-month survival is 78.5%, 71.7%, 66.6%). DSS of women with Type II EOC (red curve, *n*= 23,484; 50, 100, & 200-month survival is 50.6%, 31.7%, 22.9%). *p*-value obtained by the log rank test. The number of women remaining at different survival points is shown for Type I (blue) and Type II (red) epithelial ovarian cancers. (Type I vs. Type II: *p* < 0.001). The number of women remaining at different survival points is color-coded for each of the four categories along the *x*-axis. Vertical lines identify censored data points. (**B**). DSS of women with Type I & II EOC according to stage. Early stage (I & II) Type I EOC (blue curve, *n*= 8,527; 50, 100 & 200-month survival is 91.9%, 87.7%, 84.9%). Early stage (I & II) Type II EOC (green curve, *n*= 4,261; 50, 100 & 200-month survival is 83.9%, 71.0%, 60.3%). Late stage (III & IV) Type I EOC (red curve, *n*= 3,069; 50, 100 & 200-month survival is 47.9%, 35.3%, 28.1%). Late stage (III & IV) Type II EOC (brown curve, *n*= 16,593; 50, 100 & 200-month survival is 44.2%, 23.4%, 15.2%) (Type I vs. Type II: *p* < 0.001). *p*-value obtained by the log rank test. The number of women remaining at different survival points is color-coded for each of the four categories along the *x*-axis. Vertical lines identify censored data points.

Analysis of EOC showed that Type I DSS (blue: 50, 100 & 200-month survivals = 78.5%, 71.7%, 66.6%) is clearly superior to Type II DSS (red: 50, 100 & 200-month survivals = 50.6%, 31.7%, 22.9%, (*p* < 0.001, Figure 4A) with Type II DSS survival being 64–34% of Type I over this 200-month range. Late stage Type I EOC had a significant mortality (red curve: 50, 100 & 200-month survival = 47.9%, 35.3%, 28.1%), which was 52% to 33% of the DSS of early stage Type I (blue curve: 50, 100 & 200-month survival = 91.9%, 87.7%, 84.9%) over 50–200 months (Figure 4B) indicating that Type I EOC should not to be considered indolent because its mortality can be significant. The DSS of early stage Type II EOC (green curve, 50, 100 & 200-month survivals = 83.9%, 71.0%, 60.3%) was much better than late stage Type II EOC DSS (brown curve; 50, 100 & 200-month survivals = 44.2%, 23.4%, 15.2%) over 50–200 months (Figure 4B), indicating that late stage Type II disease is very aggressive, while early stage Type II disease has a reasonably high 200 month DSS (60.3%). Thus, as extent of disease described by stage increases, DSS becomes poorer for both Type I and II EOC.

## 4. Discussion

The important finding presented here is that the mortality of Type I late stage (III & IV) EOC is significant enough to challenge considering it as indolent. In addition, Type II early stage (I & II) EOC showed high DSS at 200 months (60.3%), indicating that when the extent of disease is limited the opportunity to overwhelm the body or “aggressiveness” is moderated. The work presented here is in agreement with recent reports on overall survival of EOC histo-types by Peres et al [27] and Lan & Yang [28]. The details of this agreement and comparisons to the work presented here are included in Table S2 of the Supplementary Material section. The work reported here is validated by the 2-tiered classification exclusively for serous EOCs with grade 3 serous carcinoma DSS inferior to grade 3 DSS of mucinous, endometrioid, clear cell carcinomas, and undifferentiated carcinomas. Expanded considerations to extent of disease (i.e., stage) revealed that the 2-tier classification for serous EOC applied only to late stage EOC since DSS of grades 2 and 3 were significantly different in early stage disease. For all the other EOC histo-types (endometrioid, clear cell, mucinous and carcinosarcoma) DSS of late stage grades 2 and 3 differed, demonstrating poorest survivals in late stage grade 3. For late stage carcinosarcomas, DSS of all grades was similarly poor and much lower than for all grades of early stage disease. Early stage undifferentiated ovarian carcinomas showed better DSS than late stage. Statistically significant breakpoints for early stage 10 year DSS (>93%, 90%, 80–87%, 68–79%, 54–65%, 44%) and late stage 10 year DSS (51–64%, 38%, 27–33%, 22–25%, <18%) were identified. Across all histo-types better DSS occurred at each grade in early stage disease over late stage disease.

With the present data set, DSS of Type I EOCs was much better than Type II. However, 33–52% decreased DSS was observed with late stage Type I over early stage Type I indicating that late stage Type I EOCs should not be considered indolent. Early stage Type II EOCs had much better DSS than late stage Type II so that the least favorable DSS was associated with extent of disease described by stage with both Type I and II EOCs.

There are several compelling reasons to examine DSS. First, although ovarian cancer is the leading cause of death for women diagnosed with EOC [29,30], among 10+ year survivors of EOC half again as many succumbed to other cancers while death due to other causes (cardiovascular disease, chronic conditions, accidents/etc) was responsible for more deaths than EOC (184%) [31]. Thus, it is important to understand the true mortality due to EOC, which is the focus of the present work. Secondly, it is important to provide oncology treatment specialists with baseline estimates of DSS so that the effectiveness of treatment against EOC *per se* can be evaluated.

The results presented here also speak to the promise of early detection by screening for both Type I & II EOC. In contrast to the speculation that screening may miss aggressive Type II EOC [32], results shown here demonstrate that if early stage Type II can be identified, improved DSS occurs. Consequently, both Type I and II EOC are bona fide targets for early detection through screening.

A significant implication of this study is regarding the balance of EOC histo-types, specifically Type I and Type II malignancies, in randomly controlled trials of cancer therapeutics. In this context, it is entirely possible that a significant treatment effect can result when the treatment group is over-weighted with histo-types that have favorable survival characteristics, or the control group is under-weighted with these same histo-types. Our survey of active treatment trials showed that Type I & II EOCs might be discernable in 6 out of 30 trials due to the availability of grade [33]. However, 13 out of 30 trials did not distinguish histological type and were open to multiple histo-types of EOC. The most straightforward approach for testing this possibility is to conduct sub-group specific analysis comparing survivals in the treatment and control groups.

This report benefits from the inclusion of a large sample of women from across the United States so that its strengths are due to the use of population level data from the SEER data set. Nevertheless, we are aware of a number of limitations. First, a substantial number of cases were excluded because information on histology, grade, stage and cause of death was missing in the SEER data. This type of incompleteness in SEER data has been recognized at the individual level [22]. Errors within SEER at the diagnosis or data entry level have been acknowledged with the understanding that in a large sample size they will not have a differential impact as they will tend to push null results, blunting rather than creating signals in the data analyses [22]. Comorbidities, like smoking status or obesity, are not reported in SEER, and while their impact cannot be ruled out, the use of DSS allows their influence toward lesser impact. Accuracy of the cause of death on death certificates has been reported to be high so that this should not impair DSS analyses in the SEER data [34,35]. SEER data does oversample individuals that are foreign-born, urban dwellers, and certain racial and ethnic groups [22]. Additionally, in our overall focus on Type I & II EOC survival, we have not isolated racial and socioeconomic disparities, Medicaid payer status, uninsured payer status, and household income, which other investigators have studied [36]. Agreement between pathologists assigning grade for SEER specimens has been reported to be best for high grade cases [37], minimizing any impact on Type I vs. II classification. Finally, the results reported here are quite consistent with two recent comprehensive studies on the overall survival of EOC histo-types [27,28] and for the first time characterize the DSS of Type I and Type II EOC. The degree to which the primary ovarian mucinous carcinoma designation is contaminated by mucinous metastatic colorectal and appendiceal carcinomas is difficult to ascertain because SEER data does not indicate when a diagnosis is made using definitive immunohistochemical stains; however, only ~7% could be expected to arise as metastases from the GI tract [38,39,40]. Presently, CAP (College of American Pathologists) protocols state that clear cell carcinomas and carcinosarcomas are not graded based on the view that there is no grading system that prognosticates the outcomes for these histo-types [41]. Thus, more recent information entered into SEER may not specify grade and, consequently, is not included in the DSS analysis for these histo-types.

## Figures and Tables

**Table 1:** EOC sub-types by stage and grade taken from KM plots.

	Stage I–II	Stage I–II	Stage III–IV	Stage III–IV
***Serous***	*n*	*10 yr Survival*	*n*	*10 yr Survival*
Grade 1	530	85.2% (81.3–88.4%)	697	51.0% (46.8–55.0%)
Grade 2	1022	73.1% (69.8–76.0%)	2983	24.6% (23.0–26.3%)
Grade 3	2032	64.8% (62.3–67.1%)	11,801	23.9% (23.1–24.7%)
***Endometrioid***	*n*	*10 yr Survival*	*n*	*10 yr Survival*
Grade 1	1759	93.4% (91.8–94.6%)	142	63.9% (54.1–72.1%)
Grade 2	2033	87.2% (85.4–88.8%)	564	38.1% (33.8–42.5%)
Grade 3	951	74.2% (70.9–77.3%)	1026	32.9% (29.8–36.0%)
***Clear Cell***	*n* =	*10 yr Survival*	*n*	*10 yr Survival*
Grade 1	48	81.1% (64.4–90.5%)	7	16.7% (0.8–51.7%)
Grade 2	284	81.4% (75.5–86.0%)	88	26.8% (17.5–37.0%)
Grade 3	902	78.3% (75.5–80.9%)	478	25.3% (21.9–28.9%)
***Mucinous***	*n*	*10 yr Survival*	*n*	*10 yr Survival*
Grade 1	1180	89.6% (87.3–91.5%)	197	32.9% (26.1–40.0%)
Grade 2	1000	84.8% (82.0–87.3%)	328	22.1% (17.6–27.0%)
Grade 3	235	72.8% (66.4–78.2%)	286	14.9% (11.2–19.2%)
***Carcinosarcoma***	*n*	*10 yr Survival*	*n*	*10 yr Survival*
Grade 1	24	100% (100–100%)	2	0.0% (0.0–0.0%)
Grade 2	13	68.4% (30.6–88.6%)	19	15.8% (3.9–34.9%)
Grade 3	107	44.2% (35.4–52.7%)	342	17.6% (14.4–21.1%)
***Undifferentiated carcinoma***	*n*	*10 yr Survival*	*n*	*10 yr Survival*
Grade 3	63	54.6% (39.7–67.3%)	213	23.7% (17.9–30.0%)

Survival is expressed with 95% confidence level. Comparisons were tested by Chi-square analysis for difference across and within grades.

**Table 2:** DSS Groupings of Early Stage EOC Subtypes Based on Grade. Breakpoints in survival were determined by Log rank test for significant difference of pairs of EOC subtype grades arranged successively. Breakpoints for statistically significant groupings: 93%, 90%, 80–87%, 68–79%, 54–65% and 44% 10-year DSS.

Early Stage					
**Stage I-II**					
Endometrioid grade 1=	Mucinous grade 1>				
Carcinosarcoma grade 1 >	**89.6% *10 yr survival***	Serous grade 1 =			
***93+% 10 yr survival***		Endometrioid grade 2=	Serous grade 2 =		
		Clear cell grade 1 =	Endometrioid grade 3 =	Serous grade 3	
		Mucinous grade 2 >	Clear cell grade 2&3 =	Undifferentiated carcinoma >	Carcinosarcoma grade 3
		***80*–*87% 10 yr survival***	Mucinous grade 3=	***54*–*65% 10 yr survival***	***44% 10 yr survival***
			Carcinosarcoma grade 2>		
			***68*–*79% 10 yr survival***		

**Table 3:** DSS Groupings of Late Stage EOC Subtypes Based on Grade. Breakpoints in survival were determined by Log rank test for significant difference of pairs of EOC subtype grades arranged successively. Breakpoints for statistically significant groupings: 51–64%, 38%, 27–33%, 22–25% and <18% 10-year DSS.

Late Stage				
**Stage III–IV**				
Endometrioid grade 1 = Serous grade 1>				
***51*–*64% 10 yr survival***	Endometrioid grade 2>			
	***38% 10 yr survival***	Endometrioid grade 3=	Mucinous grade 2=	
		Clear cell grade 2=	Serous grade 3 =	
		Mucinous grade 1= Serous grade 2>	Clear cell grade 3=	Clear cell grade 3 =
		***27*–*33% 10 yr survival***	Undifferentiated carcinoma >	Mucinous grade 3 =
			***22*–*25% 10 yr survival***	Carcinosarcoma grade 2 &3 >
				***14*–*18% 10 yr survival***

## Supplementary Materials

The following are available online at https://www.mdpi.com/2075-4418/10/2/56/s1.

## References

[1] Siegel R.L., Miller K.D., Jemal A. (2020). Cancer statistics, 2020. CA A Cancer J. Clin..

[2] Howlader N., Noone A.M., Krapcho M., Garshell J., Miller D., Altekruse S.F., Kosary C.L., Yu M., Ruhl J., Tatalovich Z. (2015). Seer Cancer Statistics Review.

[3] Torre L.A., Trabert B., DeSantis C.E., Miller K.D., Samimi G., Runowicz C.D., Gaudet M.M., Jemal A., Siegel R.L. (2018). Ovarian cancer statistics. CA A Cancer J. Clin..

[4] Goodman M.T., Yurii B., Shvetsov Y.B. (2009). Incidence of Ovarian, peritoneal, and fallopian tube carcinomas in the United States, 1995–2004. Cancer Epidemiol. Biomark. Prev..

[5] Pavlik E.J., van Nagell J.R. (2013). Early detection of ovarian tumors using ultrasound. Womens Health Lond.

[6] Committee on the State of the Science in Ovarian Cancer Research, Board on Health Care Services, Institute of Medicine, National Academies of Sciences, Engineering, and Medicine (2016). Ovarian Cancers: Evolving Paradigms in Research and Care.

[7] Crum C.P. (2009). Intercepting pelvic cancer in the distal fallopian tube: Theories and realities. Mol. Oncol..

[8] Gershenson D.M., Tortolero-Luna G., Malpica A., Baker V.V., Whittaker L., Johnson E., Follen M.M. (1996). Ovarian intraepithelial neoplasia and ovarian cancer. Obstet. Gynecol. Clin. N. Am..

[9] Kindelberger D.W., Lee Y., Miron A., Hirsch M.S., Feltmate C., Medeiros F., Callahan M.J., Garner E.O., Gordon R.W., Birch C. (2007). Intraepithelial carcinoma of the fimbria and pelvic serous carcinoma: Evidence for a causal relationship. Am. J. Surg. Pathol..

[10] Crum C.P., McKeon F.D., Xian W. (2012). The oviduct and ovarian cancer: Causality, clinical implications, and “targeted prevention”. Clin. Obstet. Gynecol..

[11] SEER Explorer: An Interactive Website for SEER Cancer Statistics [Internet]. https://seer.cancer.gov/explorer/.

[12] Seidman J.D., Kurman R.J., Ronnett B.M. (2003). Primary and metastatic mucinous adenocarcinomas in the ovaries: Incidence in routine practice with a new approach to improve intraoperative diagnosis. Am. J. Surg. Pathol..

[13] Seidman J.D., Horkayne-Szakaly I., Haiba M., Boice C.R., Kurman R.J., Ronnett B.M. (2004). The histologic type and stage distribution of ovarian carcinomas of surface epithelial origin. Int. J. Gynecol. Pathol..

[14] Gilks C.B., Ionescu D.N., Kalloger S.E., Kobel M., Irving J., Clarke B., Santos J., Le N., Moravan V., Swenerton K. (2008). Ovarian Cancer Outcomes Unit of the British Columbia Cancer Agency. Tumor cell type can be reproducibly diagnosed and is of independent prognostic significance in patients with maximally debulked ovarian carcinoma. Hum. Pathol..

[15] Meinhold-Heerlein I., Fotopoulou C., Harter P., Kurzeder C., Mustea A., Wimberger P., Hauptmann S., Sehouli J. (2016). The new WHO classification of ovarian, fallopian tube, and primary peritoneal cancer and its clinical implications. Arch. Gynecol. Obstet..

[16] Kurman R.J., Shih I.-M. (2001). Molecular pathogenesis and extraovarian origin of epithelial ovarian cancer—Shifting the paradigm. Hum. Pathol..

[17] Shih I.-M., Kurman R.J. (2004). Ovarian tumorigenesis: A proposed model based on morphological and molecular genetic analysis. Am. J. Pathol..

[18] Koshiyama M., Matsumura N., Konishi I. (2014). Recent concepts of ovarian carcinogenesis: Type I and Type II. Biomed Res. Int..

[19] Meinhold-Heerlein I., Hauptmann S. (2014). The heterogeneity of ovarian cancer. Arch. Gynecol. Obstet..

[20] Nezhat F.R., Apostol R., Nezhat C., Pejovic T. (2015). New insights in the pathophysiology of ovarian cancer and implications for screening and prevention. Am. J. Obstet. Gynecol..

[21] Soong T.R., Howitt B.E., Horowitz N., Nucci M.R., Crum C.P. (2019). The fallopian tube, “precursor escape” and narrowing the knowledge gap to the origins of high-grade serous carcinoma. Gynecol. Oncol..

[22] Duggan M.A., Anderson W.F., Altekruse S., Penberthy L., Sherman M.E. (2016). The surveillance, epidemiology, and end results (SEER) program and pathology: Toward strengthening the critical relationship. Am. J. Surg. Pathol..

[23] McGuire W.P., Hoskins W.J., Brady M.F., Kucera P.R., Partridge E.E., Look K.Y., Clarke-Pearson D.L., Davidson M. (1996). Cyclophosphamide and cisplatin compared with paclitaxel and cisplatin in patients with stage III and stage IV ovarian cancer. N. Engl. J. Med..

[24] Malpica A., Deavers M.T., Lu K., Bodurka D.C., Atkinson E.N., Gershenson D.M., Silva E.G. (2004). Grading ovarian serous carcinoma using a two-tier system. Am. J. Surg. Pathol..

[25] Vang R., Shih I.M., Kurman R.J. (2009). Ovarian low-grade and high-grade serouw carcinoma: Pathogenesis, clinicopathologic and molecular biologic features, and diagnostic problems. Adv. Anat. Pathol..

[26] Bodurka D.C., Deavers M.T., Tian C., Sun C.C., Malpica A., Coleman R.L., Lu K.H., Sood A.K., Birrer M.J., Ozols R. (2012). Reclassification of serous ovarian carcinoma by a 2-tier system: A Gynecologic Oncology Group Study. Cancer.

[27] Peres L.C., Cushing-Haugen K.L., Köbel M., Harris H.R., Berchuck A., Rossing M.A., Schildkraut J.M., Doherty J.A. (2019). Invasive epithelial ovarian cancer survival by histotype and disease stage. J. Natl. Cancer Inst..

[28] Lan A., Yang G. (2019). Clinicopathological parameters and survival of invasive epithelial ovarian cancer by histotype and disease stage. Future Oncol..

[29] Dinkelspiel H.E., Champer M., Hou J., Tergas A., Burke W.M., Huang Y., Neugut A.I., Ananth C.V., Hershman D.L., Wright J.D. (2015). Long-term mortality among women with epithelial ovarian cancer. Gynecol. Oncol..

[30] Arora N., Talhouk A., McAlpine J.N., Law M.R., Hanley G.E. (2018). Long-term mortality among women with epithelial ovarian cancer: A population-based study in british columbia, Canada. BMC Cancer.

[31] Arora N., Talhouk A., McAlpine J.N., Law M.R., Hanley G.E. (2019). Causes of death among women with epithelial ovarian cancer by length of survival post-diagnosis: A population-based study in British Columbia, Canada. Int. J. Gynecol. Cancer.

[32] Kurman R.J., Visvanathan K., Roden R., Wu T.C., Shih I.-M. (2008). Early detection and treatment of ovarian cancer: Shifting from early stage to minimal volume of disease based on a new model of carcinogenesis. Am. J. Obstet. Gynecol..

[33] Clinical Trials.gov. https://www.clinicaltrials.gov/ct2/results?cond=ovarian+cancer&term=&cntry=&state=&city=&dist=.

[34] Percy C., Stanek E., Gloeckler L. (1981). Accuracy of cancer death certificates and its effect on cancer mortality statistics. Am. J. Public Health.

[35] German R.R., Fink A.K., Heron M., Stewart S.L., Johnson C.J., Finch J.L., Yin D. (2011). Accuracy of Cancer Mortality Study Group. The accuracy of cancer mortality statistics based on death certificates in the United States. Cancer Epidemiol..

[36] Bristow R.E., Powell M.A., Al-Hammadi N., Chen L., Miller J.P., Roland P.Y., Mutch D.G., Cliby W.A. (2013). Disparities in ovarian cancer care quality and survival according to race and socioeconomic status. JNCI J. Natl. Cancer Instit..

[37] Matsuno R.K., Sherman M.E., Visvanathan K., Goodman M.T., Hernandez B.Y., Lynch C.F., Ioffe O.B., Horio D., Platz C., Altekruse S.F. (2013). Agreement for tumor grade of ovarian carcinoma: Analysis of archival tissues from the surveillance, epidemiology, and end results residual tissue repository. Cancer Causes Control..

[38] Lewis M.R., Deavers M.T., Silva E.G., Malpica A. (2006). Ovarian involvement by metastatic colorectal adenocarcinoma: Still a diagnostic challenge. Am. J. Surg. Pathol..

[39] Perrin T.J. (2016). Mucinous epithelial ovarian carcinoma. Ann. Oncol..

[40] Differentiation of Primary Ovarian Tumours from Metastatic Colorectal Carcinoma. http://e-immunohistochemistry.info/web/Differentiation_of_primary_ovarian_tumours_from_metastatic_colorectal_carcinoma.htm.

[41] College of American Pathologists Protocol Protocol for the Examination of Specimens from Patients with Primary Tumors of the Ovary, Fallopian Tube, or Peritoneum. https://documents.cap.org/protocols/cp-femalereproductive-ovary-fallopian-18protocol-1100.pdf.

