# Supplementary material for: Disease-Specific Survival of Type I and Type II Epithelial Ovarian Cancers—Stage Challenges Categorical Assignments of Indolence & Aggressiveness
Source: Diagnostics (Basel). 2020 Jan 21;10(2):56. doi: 10.3390/diagnostics10020056 (PMC7168156; doi:10.3390/diagnostics10020056)
Supplement: Supplementary file 1 [file diagnostics-10-00056-s001.pdf]

Table S1. Comparisons of Type I and II EOC.[1, 2,3,4]

	Type I		Type II	
Behavior	Indolent, 5 yr survival ~55%		Aggressive, 5 yr survival ~30%	
Continuum	cystadenoma/adenofibroma-> borderline tumors-> invasive ca		Fimbrial epithelium of fallopian tube	
		**Precursor**		**Precursor**
Endometrioid carcinoma (EC, 3-5%)	**Low grade (grade 1&2)** mutations in PTEN tumor suppressor (20%) with KRAS expression	Associated with endometriosis (19%)	**High grade (grade 3+)**	Associated with endometriosis (19%)
Clear cell carcinoma (CCC, 10-26%)	**All grades: 1,2,3** , mutations in PTEN tumor suppressor (8.3%)	Associated with endometriosis (36%)	----	----
Mucinous carcinoma (MC, 2-6%)	**All grades:1,2,3,4**	Mucinous cystadenoma; associated with endometriosis, teratoma, Brenner tumor, MBT	----	----
Serous carcinoma (SC, 73-80%)	**Low grade (grade 1, 3-5%)**	Serous cystadenoma, adenofibroma, atypical serous tumor (SBT)	**High grade (grade 2 & 3+, 70-75%)**	Ovarian surface epithelium; SCOUT-> p53 signature -> STIL/TILT->STIC
Transitional cell carcinoma (TCC), 2%/ Brenner tumor (BT, 1-3%)	**All grades:1,2,3,4**	Brenner tumor	----	----
Undifferentiated carcinoma (UC, 5%)	----	----	**All are grade 3**	?
Carcinosarcoma (CS, 1%)	----	----	**All grades:1,2,3+**	?
Genome	Not very unstable		Highly unstable	
TP53 mutation	Low (LGSC<10%)		High (HGSC ~80%)	

BRCA1/2 mutation	Low	[redacted]	Mutated, hypermethylated or dysfunctional (HGSC)	[redacted]
PTEN mutation	15-20% (EC: 20%, CCC: 8%)	[redacted]	Low	[redacted]
HNF-1 beta overexpression	90% (EC, CCC)	[redacted]	Low	[redacted]
ARIDIA mutation	40-50% (EC)	[redacted]	Not found	[redacted]
CTNNB1 mutation	30% (EC)	[redacted]	Low	[redacted]
PIK3CA	0% (grade 1 or 2 EC & CCC)	[redacted]	25% (grade 3+ EC & CCC)	[redacted]
Microsatellite instability	50% (EC, CCC)	[redacted]	8-28%	[redacted]
KRAS mutation	30-65% (EC, MC)	[redacted]	Low	[redacted]
BRAF mutation	30-65% (LGSC)	[redacted]	Low	[redacted]
HER2/neu overexpression	Low	[redacted]	HGSC: 20-67%	[redacted]
AKT overexpression	Low	[redacted]	12-30%	[redacted]
p16 inactivation	Low	[redacted]	15%	[redacted]
HLA-G overexpression	Low LGSC	[redacted]	HGSC: 61%	[redacted]
APO E overexpression	Low	[redacted]	HGSC: 66%	[redacted]
Ki67 proliferation index	10-15%	[redacted]	HGSC: 50-75%	[redacted]

Serous, endometrioid, mucinous, clear cell, transitional cell, Brenner tumor, undifferentiated carcinomas and carcinosarcomas make up the different subtypes of epithelial EOC.[5] Traditionally, EOC is thought to arise from the ovarian surface epithelium and through metaplastic transformation.[6] Recent studies have challenged this paradigm and suggest a dualistic model of EOC carcinogenesis involving Type I and Type II EOC.[2] Type I tumors behave indolently and develop from benign precursor lesions that progress towards borderline tumors and then finally to invasive tumors. These tumors are often large, unilateral, cystic, and confined to the ovary at the time of diagnosis with genomic stability. They are thought to originate from benign extraovarian lesions that embed in the ovary and undergo several mutations leading to malignant transformation. For example, low-grade serous carcinoma may develop from deposits of benign fallopian tube epithelium in the ovary, and clear cell and endometrioid carcinomas from endometriosis. Type I tumors are genetically stable with mutations in KRAS, BRAF, PTEN, PIK3CA, CTNNB1, ARID1A, and PPP2R1A. P53 mutations are rare.[7] Low-grade endometrioid tumors carry CTNNBI, PTEN, and PIK3CA mutations; low-grade mucinous tumors have mutations in

KRAS; low-grade clear cell tumors can carry PIK3CA mutations.[2] Patients with Type I tumors are younger, and there appears to be an increased risk for EOC with higher BMI.[8] Additionally, better disease-free survival has been noted in Type I tumors.[9]

In an analysis of the Prostate, Lung, Colorectal and Ovarian (PLCO) screening trial, Type I tumors had a greater likelihood of being diagnosed at early (Stage I/II) stage.[10]

In contrast, Type II tumors are unvaryingly high grade, develop rapidly, and behave aggressively, with > 75% of cases presenting in advanced stage. Type II tumors consist of high-grade serous carcinoma, high-grade endometrioid carcinoma, malignant mixed mesodermal tumors (carcinosarcomas), and undifferentiated carcinomas.[11] Extraovarian disease of Type II tumors is substantially greater with involvement of the omentum and mesentery. Ascites generally accompanies Type II EOC tumors. At the molecular level, high-grade serous carcinomas overwhelmingly carry TP53 mutations.[2] High-levels of chromosomal rearrangements have been identified resulting in genetic instability in Type II tumors. Inactivation of BRCA 1/2 by mutation or other mechanisms occurs in 40-50% of sporadic high-grade serous carcinomas.[3]

Table S2. Results from recent EOC survival analyses.

	Pavlik – this report	Peres et al[12]	Lan & Yang[13]
SEER submission period	1995-2015	2004-2014	1973-2015
Survival type	Disease-specific	Overall	Overall
Size of SEER set analyzed	35,901:<br>Serous: 21043<br>low grade: 1351<br>high grade: 19692<br>Endometrioid: 6912<br>Clear cell: 2605<br>Mucinous: 3636<br>Carcinosarcoma: 1319<br>Undifferentiated: 304	28,118:<br>Serous: 18545<br>low grade: 708<br>high grade: 17837<br>Endometrioid: 2782<br>Clear cell: 2695<br>Mucinous: 2641<br>Carcinosarcoma: 1381<br>Malignant Brenner: 74	77,658<br>Serous: 49480<br>low grade: 2755<br>high grade: 46,735<br>Endometrioid: 7460<br>Clear cell: 6214<br>Mucinous: 10612<br>Carcinosarcoma: 3613<br>Malignant Brenner: 269
Regardless of stage, best survivor outcomes	Low grade mucinous, low grade endometrioid (Fig 2A), low grade clear cell	Endometrioid & low grade serous	Low grade serous, endometrioid (Table 3), clear cell **(*did not analyze for grade, except serous**)
Localized/regional disease (stage I & II) worst survival	Carcinosarcoma	Carcinosarcoma	Carcinosarcoma
Localized/regional disease (stage I & II) worse survival within 2 yrs	Carcinosarcoma	Carcinosarcoma	Carcinosarcoma
Distant disease (stage III & IV), poor survival & similar	Grade 3 clear cell, grade 2-3 mucinous, grade 2-3 serous & all grades carcinosarcoma	Clear cell, mucinous & carcinosarcoma	Clear cell, mucinous & carcinosarcoma
Distant disease (stage III & IV), poor survival & similar within 2 yrs (to p 62 end para 2)	Grade 3 clear cell > grade 2-3 mucinous> grade 2-3 serous > all grades carcinosarcoma	Clear cell, mucinous & carcinosarcoma	Clear cell, mucinous & carcinosarcoma
Localized disease (stage I): 1 & 5 yr survivals	All low grade histotypes >80%, except carcinosarcoma (5yr: 72.2%, 67.9-76.0%)	All histotypes >80%, except carcinosarcoma (5yr: 70.7%, 56.3-81.1%)	All histotypes >80%, except carcinosarcoma (5yr: 65.4%)
Regional disease (stage II): 1 yr survivals	All histotypes >80%, except grade 3 carcinosarcoma	All histotypes >80%	All histotypes >80%, except carcinosarcoma
Regional disease (stage II): 5 yr survivals	All histotypes <80%, except grade 1 & 2endometrioid & grade 1 & 2 clear cell & grade 1 &2 mucinous & grade 1 serous	All histotypes <80%, except low grade serous & endometrioid	All histotypes <80%, except low grade serous & endometrioid

Regional disease (stage II): 5 yr survivals	Worst outcomes for carcinosarcoma	Worst outcomes for carcinosarcoma (38.6%, 32-45.1%)	Worst outcomes for carcinosarcoma (40.4%)
Regional disease (stage II): 10 yr survivals	Worst outcomes for carcinosarcoma	Worst outcomes for carcinosarcoma (29%, 22.4-35.9%)	Worst outcomes for carcinosarcoma (30.5%)
Across all survival times & regardless of stage: best outcome	Endometrioid> low grade serous	Low grade serous	Endometrioid> low grade serous
Distant disease (stage III & IV), 1 yr survival	<65% --- mucinous (64.9%, 64.3-65.6%)	<40% --- mucinous (37.9%, 33.9-41.8%)	<50% --- mucinous (42%)
Distant disease (stage III & IV), 1 yr survival	Clear cell (74.3%, 73.3-75.6%), carcinosarcoma (58.8%, 56.7-60.8%)	Clear cell (63.3%, 58.5-67.6%), carcinosarcoma (60%, 56.8-63%)	Clear cell (65%), carcinosarcoma (58%)
Distant disease (stage III & IV), 1 yr survival	All other histotypes >80%	All other histotypes >80%	All other histotypes >80%
Distant disease (stage III & IV), 5 yr survival	Most histotypes <35% & clear cell, carcinosarcoma & mucinous <35%	Most histotypes <35% & clear cell, carcinosarcoma & mucinous <22%	Most histotypes <35% & clear cell, carcinosarcoma & mucinous <22%
Distant disease (stage III & IV), 10 yr survival	clear cell, carcinosarcoma & mucinous <35%	clear cell, carcinosarcoma & mucinous <22%	clear cell, carcinosarcoma & mucinous <22%
Distant disease (stage III & IV), 5-10 yr survival	All other histotypes survival continued to decline	All other histotypes survival continued to decline	All other histotypes survival continued to decline
Distant disease (stage III & IV), best survival, 10 yr survival	Endometrioid (53.8, 52.7-54.8%)	Low grade serous (37.3%, 29-45.7%)	Low grade serous (40.7%), Endometrioid (34.4%)
Localized/regional disease (stage I & II) all survival periods	Risk of mortality:<br>Endometrioid & low grade serous better than high grade serous	Risk of mortality:<br>Endometrioid & low grade serous better than high grade serous	Risk of mortality:<br>Endometrioid & low grade serous better than high grade serous
Localized disease (stage I), 1-4 yrs survival	Risk of mortality:<br>high grade serous better than carcinosarcoma	Risk of mortality:<br>high grade serous better than carcinosarcoma	Risk of mortality:<br>high grade serous better than carcinosarcoma
Localized disease (stage I), 4-10 yrs survival ?	Relative to high grade serous HR of all histotypes <1.<br>Cumulative survivals for high grade serous ~ carcinosarcoma	Relative to high grade serous HR of all histotypes <1.<br>Cumulative survivals for high grade serous > carcinosarcoma	Risk of mortality:<br>high grade serous better than carcinosarcoma
Localized/regional disease (stage I & II), 4-10 yrs survivals	Relative to high grade serous HR of all histotypes <1.<br>Cumulative survivals for high grade serous ~ carcinosarcoma	Relative to high grade serous HR of all histotypes <1.<br>Cumulative survivals for high grade serous > carcinosarcoma	Cumulative survivals for high grade serous > carcinosarcoma

Distant disease (stage III & IV), 1-2 yrs survival	Cumulative survivals for high grade serous > mucinous, clear cell & carcinosarcoma	Cumulative survivals for high grade serous > mucinous, clear cell & carcinosarcoma	Cumulative survivals for high grade serous > mucinous, clear cell & carcinosarcoma
Distant disease (stage III & IV), 1 yr survival	Mucinous HR 1.51, 1.45-1.56 relative to high grade serous	Mucinous HR 3.87, 3.45-4.34 relative to high grade serous	Mucinous HR 2.083, 1.97-2.197 relative to high grade serous
Most favorable outcomes	Low grade serous & endometrioid regardless of stage & grade 1 & 2 clear cell	Low grade serous & endometrioid regardless of stage	Low grade serous & endometrioid regardless of stage
Strikingly high mortality	Carcinosarcoma & distant stage mucinous and clear cell (1-2 yrs)	Carcinosarcoma & distant stage mucinous and clear cell (1-2 yrs)	Carcinosarcoma & distant stage mucinous and clear cell (1-2 yrs)
All stages of high grade serous	Highest mortality 4 or more years after diagnosis	Highest mortality 4 or more years after diagnosis	Highest mortality 4 or more years after diagnosis, except for carcinosarcoma

1. Kurman RJ, Shih I-M. Molecular pathogenesis and extraovarian origin of epithelial ovarian cancer – Shifting the paradigm. Human Pathology. 2001;42(7):918-931. doi: 10.1016/j.humpath.2011.03.003

2. Koshiyama M, Matsumura N, Konishi I. Recent concepts of ovarian carcinogenesis: Type I and Type II. Biomed research international. 2014;2014:934261. doi: 10.1155/2014/934261.

3. Willner J, Wurz K, Allison KH, Galic V, Garcia RL, Goff BA, Swisher EM. Alternate molecular genetic pathways in ovarian carcinomas of common histological types. Hum Pathol. 2007;38(4):607-13. DOI: 10.1016/j.humpath.2006.10.007.

4. Kurman RJ, Visvanathan K, Roden R, Wu TC, Shih I-M. Early detection and treatment of ovarian cancer: shifting from early stage to minimal volume of disease based on a new model of carcinogenesis. American journal of obstetrics & gynecology. 2008;198(4):351-356. doi: 10.1016/j.ajog.2008.01.005.

5. Torre LA, Trabert B, DeSantis CE, Miller KD, Samimi G, Runowicz CD, Gaudet MM, Jemal A, Siegel RL. Ovarian Cancer Statistics, 2018. CA: a cancer journal for clinicians. 2018; 68: 284-296. doi: 10.3322/caac.21456.

6. Crum CP, McKeon FD, Xian W. The oviduct and ovarian cancer: causality, clinical implications, and "targeted prevention". Clin Obstet Gynecol. 2012 Mar;55(1):24-35. doi: 10.1097/GRF.0b013e31824b1725.

7. Gershenson DM, Tortolero-Luna G, Malpica A, Baker VV, Whittaker L, Johnson E, Follen Mitchell M. Ovarian intraepithelial neoplasia and ovarian cancer. Obstet Gynecol Clin North Am. 1996 Jun;23(2):475-543.

8. Crum CP. Intercepting pelvic cancer in the distal fallopian tube: theories and realities. Mol Oncol. 2009; 3(2):165-170. doi: 10.1016/j.molonc.2009.01.004.

9. Skirnisdottir I, Seidal T, Akerud H. Differences in Clinical and Biological Features Between Type I and Type II Tumors in FIGO Stages I-II Epithelial Ovarian Carcinoma. International journal of gynecological cancer. 2015;25(7): 1239-47. doi: 10.1097/IGC.0000000000000484.

10. Terada KY, Ahn HJ, Kessel B. Differences in risk for type 1 and type 2 ovarian cancer in a large cancer screening trial. Journal of gynecologic oncology. 2016;27(3): e25. doi: 10.3802/jgo.2016.27.e25.

11. Kindelberger DW, Lee Y, Miron A, Hirsch MS, Feltmate C, Medeiros F, Callahan MJ, Garner EO, Gordon RW, Birch C, Berkowitz RS, Muto MG, Crum CP. Intraepithelial carcinoma of the fimbria and pelvic serous carcinoma: Evidence for a causal relationship. Am J Surg Pathol. 2007;31(2):161-9. DOI: 10.1097/01.pas.0000213335.40358.47.

12. Peres LC, Cushing-Haugen KL, Köbel M, Harris HR, Berchuck A, Rossing MA, Schildkraut JM, Doherty JA. Invasive Epithelial Ovarian Cancer Survival by Histotype and Disease Stage. J Natl Cancer Inst. 2019; 111(1):60-68. doi: 10.1093/jnci/djy071.

13. Lan A, Yang G. Clinicopathological parameters and survival of invasive epithelial ovarian cancer by histotype and disease stage. Future Oncol. 2019; 15(17):2029-2039. doi: 10.2217/fon-2018-0886. Epub 2019 May 29.
